# A review on β-cyclodextrin-based self-healable supramolecular systems: mechanisms and biomedical applications

**DOI:** 10.1039/d6ra00694a

**Published:** 2026-03-31

**Authors:** Nahid Sultana, Shukanta Bhowmik, Khadija Bilkis, Mofassel Hossen Akash, Fariha Afrose, Kaniz Fatema, Khodeja Afrin, Md. Shafiul Islam, Md. Tanvir Hossain, Fataha Nur Robel, Md. Ashraful Alam

**Affiliations:** a Department of Applied Chemistry and Chemical Engineering, Noakhali Science and Technology University Noakhali-3814 Bangladesh nahidsultana@nstu.edu.bd +880-1935470356; b Department of Applied Chemistry and Chemical Engineering, University of Dhaka Bangladesh

## Abstract

Supramolecular self-healing materials have attracted significant interest due to their capacity for autonomous damage repair, which enhances their durability and functional performance. This review examines the design strategies and recent advances in self-healing systems driven by host–guest interactions, with a particular focus on cyclodextrin (CD)-based architectures. β-Cyclodextrin (β-CD), owing to its biocompatibility and strong ability to form inclusion complexes, serves as a crucial building block in the design of host–guest-based self-healing systems. These self-healable materials have garnered significant attention owing to their potential applications in various fields, specifically biomedical applications. This review highlights the recent advancements in β-CD-based supramolecular materials, focusing on their unique host–guest (HG) interactions that result in the emergence of their self-healing properties. We discuss β-CD and its ability to form inclusion complexes with a range of guest molecules, which promotes reversible interactions that are critical for self-healing mechanisms. The synthesis methods, structural design, and influence of environmental conditions on the healing efficiency of these materials are critically analyzed. Furthermore, we explore the performance of β-CD-based systems in practical applications and provide insights into future research directions aimed at enhancing the robustness and applicability of these materials. Our findings underscore the potential of β-CD-based supramolecular networks as versatile platforms for the development of advanced materials with reliable self-healing capabilities.

## Abstract 2

Supramolecular self-healing materials have attracted significant interest due to their capacity for autonomous damage repair, which enhances their durability and functional performance.

## Graphical abstract

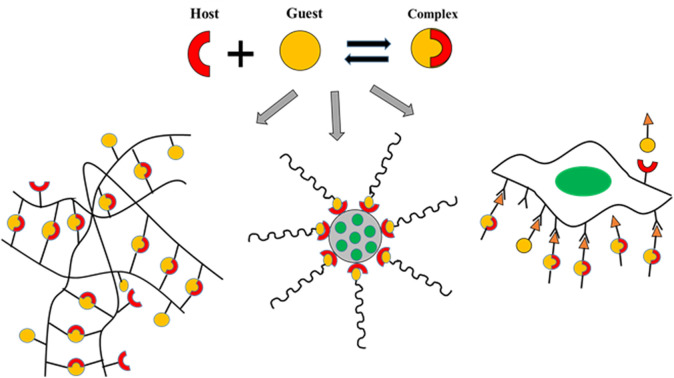

## 1. Introduction

Supramolecular polymers are formed through partially or entirely non-covalent interactions, in contrast to conventional polymers, which are produced *via* covalent bonding between monomers. Unlike traditional polymers, these non-covalently assembled supramolecular are reversible in nature, allowing them to be easily disassembled and reformed in response to changes in the local chemical environment. This special reversibility makes supramolecular polymers simple to process, fabricate, and recycle. They have tunable or shape-memory qualities, are stimuli responsive, and are self-healing.^1–10^ The ability of supramolecular polymers to form networks through noncovalent interactions allows them to self-heal after injury. There are different noncovalent interactions, such as metal–ligand coordination, ionic bonding, hydrogen bonding, and π–π interactions, and all of them have definite bond strengths and unique benefits.^11^

CDs are the water-soluble cyclic oligomers of d-(+)-glucose units that are bound to each other through α-1,4-glucose bonding. They are produced by the enzymatic processing of starch, which ensures their availability and biocompatibility, in contrast to other synthetic host molecules like crown ethers,^12^ calixarenes,^13^ cucurbiturils,^14^ and pillar[*n*]arenes.^15–17^ CDs can be easily functionalized to make hydrogels.^18–20^ Harada has made significant contributions to this field as the pioneer of CD-based supramolecular polymers.^21–25^ Harada has studied the catalytic performance of CD-modified polymers in the hydrolysis of ester compounds for 15 years, starting in 1976.^26^ Harada's studies on CD-based supramolecular polymers and associated self-assembled structures have advanced significantly since the 1990s.^27,28^ The first reports of supramolecular gels containing CDs were published in the early 1990s. The compound formed between α-CD and poly-ethylene glycol (PEG) was initially reported by Harada and Kamachi. The sol–gel transition between high molecular weight PEG and α-CD in an aqueous solution during the formation of an HG complex was examined in 1994.^29^ The interaction between the host and guest occurs when the guest moiety is physically inserted into the host moiety, representing a form of non-covalent interaction.^30^ Networks formed through these interactions can demonstrate the ability to self-repair reversibly. HG interactions have garnered significant interest and have been extensively utilized in the design of self-healable materials.

**Fig. 1:**
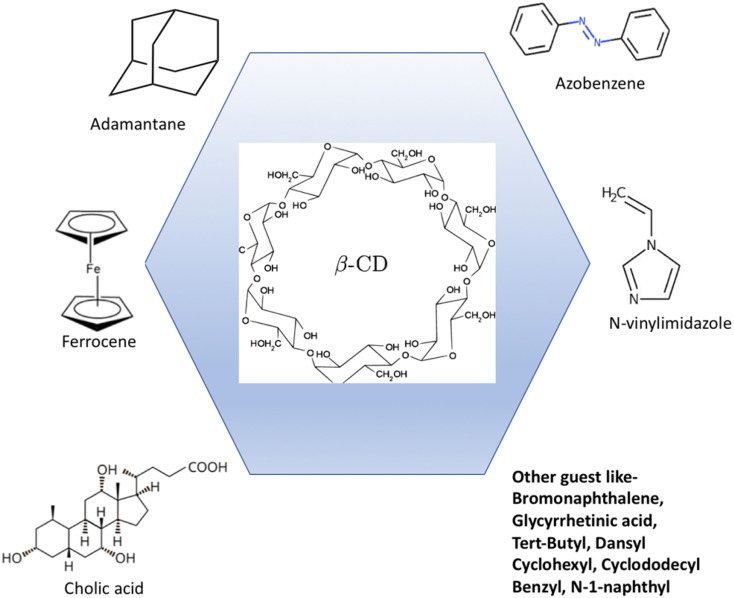
Host–guest interactions between β-CD and different guest molecules.

With an emphasis on the past ten years, we have compiled the synthesis and use of supramolecular self-healing materials using HG complexation techniques based on β-CD in this review. Different synthetic techniques can be developed to generate HG complexes, each with distinct properties and applications, by altering host and guest monomers and polymers. Because of their biocompatibility, availability, ease of modification, ability to form β-CD complexes with various guest molecules, and reversible nature, CD-based HG interactions with various guest molecules that can fit inside the β-CD cavity have drawn the attention of numerous researchers among various noncovalent interactions. Every guest molecule that enters the CD cavity gives supramolecular self-healing materials special characteristics. For instance, adamantane (AD), ferrocene (Fc), azobenzene (Azo), *N*-vinylimidazole (NV) and cholic acid (CA), in that order, result in supramolecular self-healing materials with excellent stability, electrochemical sensitivity, light sensitivity, and biocompatibility. Additionally, there are numerous uses of β-CD-based self-healing materials in biomedical,^31–33^ 3D/4D printing,^34^ coating,^35,36^ and stimuli-responsive systems^37^ as well as drug delivery^38,39^ and cancer therapy.^40^ This review systematically examines the diverse strategies employed to fabricate HG supramolecular self-healing materials based on β-CD and a variety of guest molecules, including AD, Azo, Fc, NV, CA, and related compounds. Particular emphasis is placed on the underlying HG interaction mechanisms and the emerging biomedical applications of β-CD-based supramolecular self-healing systems (Fig. 1).

## 2. β-CD as a preferred host in self-healable supramolecular polymers

CDs are cyclic oligosaccharides composed of d-glucopyranose units arranged in a truncated cone-shaped (toroidal) structure, and they are broadly classified into three parent types (α-, β-, and γ-CD) containing 6, 7, and 8 glucose units, respectively.^41^ The difference in the number of glucopyranose units determines their internal cavity diameter and physicochemical properties, thereby governing their inclusion capacity and application potential. α-CD, with a cavity diameter of approximately 4.7–5.3 Å, is suitable for encapsulating small, linear, or low molecular-weight hydrophobic molecules, such as fatty acids and aliphatic hydrocarbons. β-CD, consisting of seven glucose units and possessing an intermediate cavity diameter of approximately 6.0–6.5 Å, accommodates a wide range of aromatic and heterocyclic compounds and is the most extensively utilized CD despite its relatively low aqueous solubility. γ-CD, composed of eight glucose units, exhibits the largest cavity (approximately 7.5–8.3 Å) and the highest water solubility among the native CDs, enabling encapsulation of bulky molecules, such as steroids and certain vitamins.^42^ All CDs share a hydrophilic outer surface due to the presence of primary and secondary hydroxyl groups, and a relatively hydrophobic inner cavity, which is lined by glycosidic oxygen and hydrogen atoms, facilitating the formation of non-covalent inclusion complexes with lipophilic guest molecules in aqueous environments.^43^

The mechanism of inclusion complex formation is primarily governed by hydrophobic effects and other weak intermolecular interactions rather than covalent bonding. In an aqueous solution, the hydrophobic cavity of CDs is occupied by energetically unfavorable water molecules; upon guest inclusion, these high-energy water molecules are displaced into the bulk solvent, resulting in an entropic gain that thermodynamically favors complex formation. Additional stabilization arises from van der Waals forces, hydrogen bonding, dipole–dipole interactions, and electrostatic interactions.^44,45^ The formation of CD–guest complexes is a dynamic and reversible equilibrium process in which guest molecules continuously associate with and dissociate from the host cavity. The stability of these complexes depends strongly on the geometric complementarity and physicochemical compatibility between the host and guest; neutral and moderately hydrophobic molecules typically display higher affinity, while highly polar or ionized species exhibit reduced inclusion efficiency. Environmental factors such as temperature and solvent composition also influence complex stability; elevated temperatures generally decrease the binding affinity, while the presence of organic co-solvents (ethanol) weakens the hydrophobic driving force and reduces encapsulation efficiency.^46,47^

Despite their versatility, CDs exhibit inherent limitations, including strict cavity size constraints, limited complexation with highly hydrophilic or bulky molecules, and possible stoichiometric variability.^48,49^ Among the three CDs, β-CD is the most widely employed due to its optimal balance between cavity size, binding strength, structural rigidity, availability, and cost-effectiveness. Its intermediate cavity provides excellent steric complementarity with many hydrophobic drug molecules, particularly aromatic compounds, resulting in relatively stable inclusion complexes.^50^ Structurally, β-CD possesses a complete belt of secondary hydroxyl hydrogen bonds that confers conformational rigidity, enhancing complex stability through stronger van der Waals and hydrogen-bonding interactions compared with α- and γ-CD. In self-healable supramolecular polymers, where reversible host–guest interactions act as dynamic crosslinks, the binding affinity must be sufficiently strong to maintain mechanical integrity yet sufficiently labile to allow dissociation and reformation under mild conditions.^51–53^ β-CD typically exhibits an association with common hydrophobic guests, such as adamantane or ferrocene derivatives, which is considered optimal for dynamic network formation. In contrast, α-CD often forms complexes that are too weak to provide adequate structural stability, while the larger cavity of γ-CD may lead to less constrained or multi-guest inclusion, resulting in reduced crosslinking precision. The favorable balance of thermodynamic stability and kinetic reversibility in β-CD-based host–guest systems underpins its predominant role in the design of self-healing supramolecular materials and advanced biomedical applications.

## 3. β-CD-based self-healable supramolecular materials

### 3.1 Self-healable materials based on β-CD–AD complexation

Supramolecular materials incorporating CD and AD are synthesized by combining host and guest polymers using planetary ball milling techniques through noncovalent bonds, which enhance the toughness and flexibility of polymeric materials by leveraging their reversible characteristics to prolong their lifespan. The toughness of these supramolecular materials produced *via* ball milling is approximately 2 to 5 times greater than that of those created using the traditional casting method because ball milling facilitates the mixing of host and guest polymers at the nanoscale, thereby enabling self-healing and recycling capabilities. Notably, these materials preserve their mechanical properties even after multiple ball milling cycles. They are suitable for use as self-healing bulk materials and coatings while maintaining the transparency of the underlying substrate. Additionally, fractured segments of the materials can be rejoined within 10 min, and scars on the coating vanish within seconds at 60 °C, thus exhibiting scratch resistance due to their robust mechanical properties.^54^

**Fig. 2:**
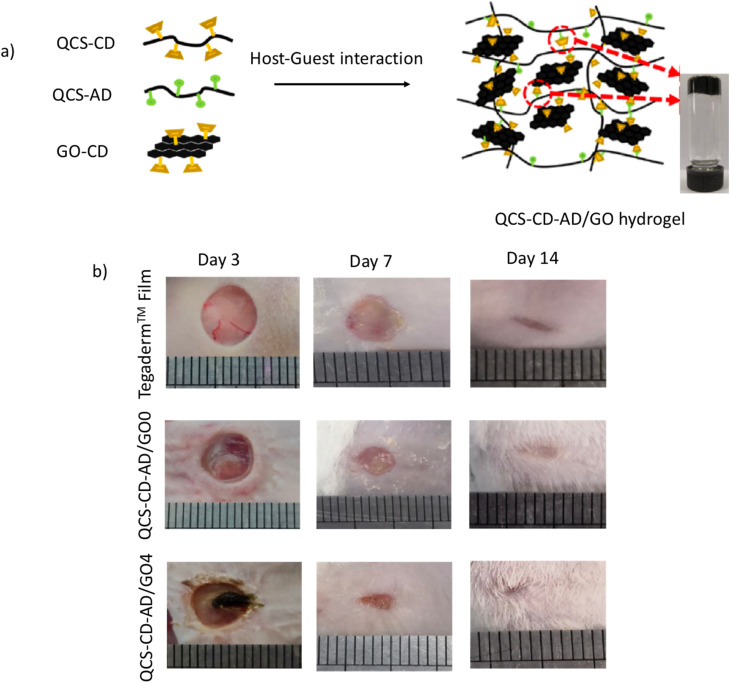
(a) Preparation of QCS–CD–AD/GO supramolecular hydrogel through HG interactions, (b) wound healing properties of QCS–CD–AD/GO and Tegaderm™ film; reproduced from ref. 56 with permission from Elsevier,^56^ copyright 2026.

The development of multifunctional injectable self-healing supramolecular hydrogels with conductive and photothermal properties for wound healing has not been previously reported. Hydrogel dressings exhibiting excellent biocompatibility and capacity to maintain a moist environment at the wound site hold significant promise for clinical use and have been developed. A series of antibacterial, injectable, self-healing, and conductive supramolecular hydrogels were created through HG interactions utilizing quaternized chitosan-*graft*-CD (QCS–CD), QCS-*graft*-AD (QCS–AD), and graphene oxide (GO)-*graft*-CD (GO–CD) polymer solutions by Zhang *et al.*^55^ These hydrogels leverage the strong antibacterial properties of QCS and the photothermal capabilities of reduced graphene oxide (rGO). The resulting supramolecular hydrogel dressings demonstrate conductivity comparable to that of human skin, exhibit rapid self-healing characteristics, and possess significant antibacterial efficacy against *E. coli*, *S. aureus* (Gram negative and Gram positive, respectively), and multi-drug resistant bacteria, including methicillin-resistant *Staphylococcus aureus* (MRSA). The QCS–CD–AD/GO4 variant achieves an optimal balance of antibacterial activity, cell proliferation, and hemo-compatibility. In comparison to commercial dressings, such as the Tegaderm™ film and the QCS–CD–AD/GO variant, the QCS–CD–AD/GO hydrogel markedly enhances the *in vivo* healing process after healing for 7 days, and after 14 days, the wounds treated with the hydrogel are completely healed and are fully covered with newly grown hair in certain mice (Fig. 2). Thus, these antibacterial, conductive, and self-healing supramolecular hydrogels represent promising biomaterials for wound dressings aimed at full-thickness skin repair.

**Fig. 3:**
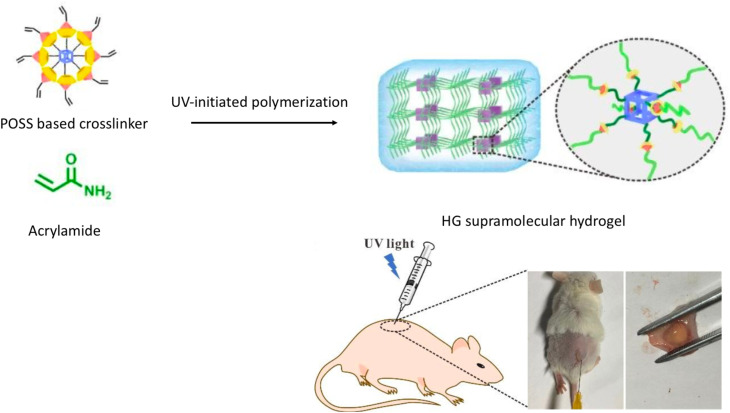
Preparation scheme of the HGP hydrogel and its *in situ* biocompatibility properties; reproduced from ref. 57 with permission from the Royal Society Chemistry,^57^ copyright 2026.

A planned layout of a star-shaped POSS-containing supramolecular crosslinker has been created to make HG hydrogels that are more flexible, have better mechanical properties, and are very compatible with living things. The HG supramolecular crosslinker based on POSS (HGP) was established through the interaction between octa-CD-modified polyhedral oligomeric silsesquioxane (OCDPOSS) and acrylamide-conjugated AD (AD-AAm) *via* UV-initiated polymerization. POSS, recognized as the smallest uniform cage-like silica nanoparticle with a robust and stable cubic core, has been extensively utilized to enhance the mechanical characteristics of hybrid materials. Cell culture studies indicate that these biocompatible hydrogels are promising candidates for applications in tissue engineering and for the sustained release of hydrophobic drugs, such as dexamethasone, for at least 14 days at the site of injection (Fig. 3).^57^

A flexible and porous fabric for electromagnetic interference (EMI) shielding has been developed by Chen *et al.*,^58^ featuring a combination of effective EMI absorption capabilities and self-healing properties. This self-healing EMI shielding fabric demonstrates significant potential for both military and civilian applications. This feature was achieved by creating aligned carbon nanotube (CNT) and poly(2-hydroxyethyl methacrylate) (PHEMA) non-woven fabric through a process of magnetic-field-assisted electrospinning and cross-stacking the aligned fabric. The alignment of the CNTs and the fabric's porosity allows for effective EMI absorption, even with a minimal CNT loading of 0.17 wt%. The EMI shielding effectiveness of the sample, which consisted of 16 stacked layers with a thickness of 2 mm, reached 20.42 dB at a frequency of 11.3 GHz, providing a shielding capability of 99.99% against electromagnetic waves. The interaction between the CD and the adhesive matrix not only facilitates automatic adhesion of the fabric layers but also enables self-healing of scratches in a 100% humidity environment. Following the self-healing process, the EMI shielding performance can be restored to 90.86.

The HG interactions within thermoplastic polyurethane (TPU) play a crucial role in stress distribution, thereby enhancing both its mechanical and self-healing properties. The pursuit of materials with high toughness and self-healing capabilities has emerged as a primary objective in material science. The TPU modified through HG interactions holds significant potential for future applications in industrial materials. Through HG interactions, TPU was enhanced to improve its mechanical properties and self-healing performance. The TPU-modified HG interactions were synthesized using step-growth bulk polymerization involving hexamethylene diisocyanate (HDI), and tetraethylene glycol (TEG), along with HG interactions between permethylated amino β-CD (PMeAm-β-CD) and AD-amine (ADAm). The TPU sample containing 10 mol% HG units [HG(10)] exhibited the highest rupture stress (11 MPa) and a fracture energy approximately 50 times higher than that of the non-functionalized TEG-based TPU. Furthermore, the damaged HG(10) demonstrated an impressive 87% recovery after being heated for 7 min at 80 °C, while a completely severed HG(10) achieved 80% recovery after 60 min of reattachment at the same temperature.^59^

An innovative hydrogel was developed through non-covalent HG interactions between β-CD-modified hyaluronic acid (HA-CD) and 4-arm-PEG-AD (4-arm-PEG-AD). The use of a multi-armed monomer facilitated an increase in the number of functional groups while minimizing steric hindrance, thereby enhancing the efficiency of HG interactions. The hydrophobic cavities of β-CD allowed for the incorporation of insoluble dexamethasone. The resulting hydrogels demonstrated remarkable self-healing capabilities. By incorporating 4-arm-PEG-AD, it was able to modulate the mechanical strength, swelling rate, and release profile of dexamethasone. These novel hydrogels significantly enhanced the therapeutic efficacy of dexamethasone in the context of burn wound healing. The therapeutic impact of the drug-loaded hydrogel revealed that after 2 weeks, the wound closure rate reached 85.09%. Thus, these hydrogels show considerable promise for the direct, convenient, and effective delivery of hydrophobic drugs, thereby improving their therapeutic outcomes.^60^

**Fig. 4:**
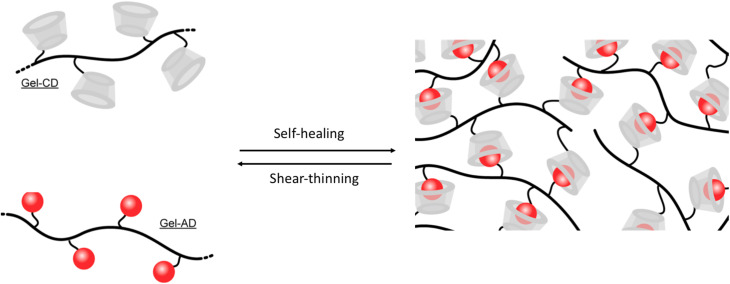
Self-healing and shear-thinning hydrogel prepared by the reaction of modified CD and AD gel; reproduced from ref. 61 with permission from John Wiley and Sons,^61^ copyright 2026.

The addition of CD and AD groups to the gelatin backbone was prepared by Sisso *et al.*,^61^ (Fig. 4), which facilitates the formation of physically cross-linked hydrogel biomaterials with adjustable mechanical characteristics. The rapid and reversible nature of CD, AD, and HG interactions allows for the creation of distinctive materials that are injectable and possess the ability to self-heal repeatedly. Gelatin is known for its biocompatibility and biodegradability, and it does not elicit an immune response. However, one limitation of gelatin is that it remains in a liquid state at room temperature, making it unsuitable as a stable scaffold for *in vivo* applications. To address this issue, gelatin was functionalized with Hg moieties (β-CD and AD) to enhance stability, improve mechanical properties, and slow down degradation. A solid-like gel was achieved with a Gel-AD to Gel-CD ratio of 2 : 1, exhibiting a storage modulus (*G*′) of approximately 400 Pa and a loss modulus (*G*″) of around 10 Pa. The stem cells encapsulated within the gels were shielded from shear forces during injection, resulting in a viability rate of approximately 95%, which is significantly higher than that of cells injected directly as suspensions.

**Fig. 5:**
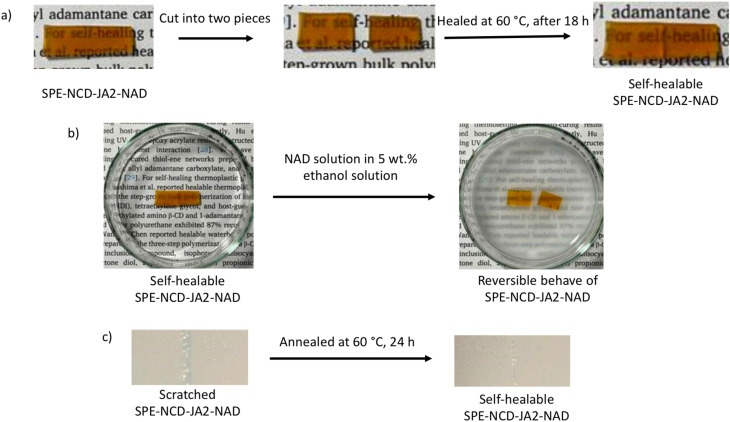
Self-healing properties of SPE-NCD-JA2-NAD under different conditions (a–c); reproduced from ref. 62 with permission from Springer Nature,^62^ copyright 2026.

Through thermal curing reactions, epoxy–amine networks incorporating CD/AD HG structures were synthesized in 2021 by Sugane's group by reacting sorbitol polyglycidyl ether (SPE) with aminated β-CD (NCD), Jeffamine® ED-600 (JA), and 1-adamantylamine (NAD). These networks exhibited self-healing properties when subjected to mild heat treatment in a non-aqueous environment. This study primarily examined the effect of JA, which enhances reversible HG inclusion complexation by lowering the glass transition temperature (*T*_g_) of the epoxy network. When two cut sections of the film were placed in contact with the cross-sectional cut at 60 °C for 18 h, they adhered to form a self-healed film of SPE-NCD-JA2-NAD. Although the two pieces of SPE-NCD-JA2-NAD bonded after just a few minutes at 60 °C, they achieved significant tensile strength after 18 h. Additionally, when the SPE-NCD-JA2-NAD self-healable film was immersed in a 5 wt% NAD solution in ethanol, the adhered pieces separated at the contact interface without any external force after 1 min. Furthermore, when this film was scratched with a cutter knife and subsequently annealed at 60 °C for 24 h, it demonstrated similar properties. In contrast, other films like SPE-NCD, SPE-NCD-JA2, and SPE-JA2-NAD, lacking both NCD and NAD components, did not exhibit self-healing capabilities under the same conditions, indicating that the presence of both NCD and NAD is essential for achieving self-healing functionality (Fig. 5).^62^

Polydimethylsiloxane (PDMS) is regarded as highly promising due to its biocompatibility. The self-healable PDMS-Me-β-CD–AD was synthesized through ring-opening reactions involving epoxy-modified PDMS with methylated β-CD (Me-β-CD) and AD-modified PDMS (AD-PDMS) by Yoshida *et al.* in 2023.^63^ The modified Me-β-CD and AD acted as supramolecular cross-links, improving the mechanical characteristics, with an increased presence of Me-β-CD and AD leading to a higher Young's modulus. When the sample was divided into two pieces, the PDMS-Me-β-CD–AD-(5) was promptly rejoined. Following a 24 h storage period at 25 °C, PDMS-Me-β-CD–AD-(5) demonstrated self-healing capabilities. The self-healed PDMS-Me-β-CD–AD-(3) achieved a healing efficiency of 30% in terms of fracture stress.

A hydrogel that is both self-healable and conductive was developed as a coating in 2023 by Park *et al.*,^64^ emphasizing HG chemistry. This innovation involves HG complexation between β-CD derivatives and AD derivatives to facilitate self-healing capabilities (Table 1). The hydrogel was synthesized using sodium poly(styrene sulfonate) (PSS-Na) combined with the HHG complexes (PSSNa-β-CD–AD(*x*)) and poly(3,4-ethylenedioxythiophene) (PEDOT) with the HG complexes (PEDOT-β-CD–AD(*y*, *z*)), where *x*, *y*, and *z* are the molar% of β-CD and AD in PSSNa-β-CD–AD(*x*) and PEDOT-β-CD–AD(*y*, *z*), respectively. The self-healing properties of the PSSNa-β-CD–AD(*x*)/PEDOT-β-CD–AD(*y*, *z*) system were examined using confocal laser scanning microscopy. It was found that PSSNa-β-CD–AD(*x*) played a crucial role in achieving effective self-healing when *x* and *y* were set to 0 and 2, respectively, and the wound exhibited partial self-healing although some areas showed an increase in cross-sectional area. In contrast, when *x* and *y* were set to 2 and 0, the damage was completely healed. The self-healing efficiency in a humid environment was significantly influenced by PSSNa-β-CD–AD(*x*) due to its hydrophilic nature.

**Table 1:** Reports representing different HG interactions between β-CD and AD, showing self-healing properties

Host	Guest	Method	Healing time	Healing efficiency	Application	Ref.
Poly(ethylenimine) modified with β-CD (PEI-β-CD)	Poly(acrylic acid) modified with AD (PAA-AD)	Layer-by-layer (LbL)	In the presence of water, <30 min	—	Acid-resistant multilayer film	65
β-CD-modified TiO_2_ nanoparticles	Hydroxyethyl methacrylated-adamantine (HEMA-AD)	Polymerization	—	—	UV-blocking coating	66
β-CD-modified Al_2_O_3_ nanoparticles	Hydroxyethylmethacrylate-AD (HEMA-AD)	Copolymerization	In the presence of water, after 2 h	85%	—	67
Amphiphilic polycaprolactone ε-β-CD, HA-β-CD macromer	AD-PEG-AD	LbL	—	—	Co-delivery of small molecules and chondrogenic growth factors	68
PEI-β-CD	PAA-AD	—	<30 min after addition of water	—	Metal ion sensors for coating as well as antibacterial purposes	69
Poly-β-CD (PCD)	*N*,*N*-Dimethyl-1-adamantanamine	—	<30 min	—	Drug carrier	70
β-CD modified cadmium telluride (CdTe)	HEMA-AD	*In situ* polymerization	<5 min in the presence of water	—	Reusable xerogel	71
Cationic β-CD oligomer allyl ether [C(β-CD-OM)AE]	1-Adamantyl acrylate	Polymerization	—	84% and <24 h	Controlled drug delivery	72
β-CD nanogels	Photolabile AD	—	<10 min	At room temperature, 65%, after 5 min	Self-repairing elastomer	73
PCD	AA-AD-AAc	Copolymerization	—	70%, after 2 h, under humid and hot conditions	Shape-memory hydrogel	74
β-CD modified with isocyanatoethyl (β-CD-AOI2)	2-(2-(2-(2-(Adamantyl-1-oxy)ethoxy)ethoxy)ethanol acrylate) (A-TEG-AD)	Polymerization	1 h	—	—	75
β-CD-modified graphene	AD glycol diglycidyl ether	—	24 h	—	Coating	76
PEI-β-CD	PAA-AD	LbL	30 min, under strong light	—	Biocompatible self-repairing coating	77
Allylated β-CD (ACD)	Allyl 1-adamantanecarboxylate (AAD)	Photo-polymerization	—	85%	Electronic/electric and coating	78

### 3.2 Self-healable materials based on β-CD–Azo complexation

**Fig. 6:**
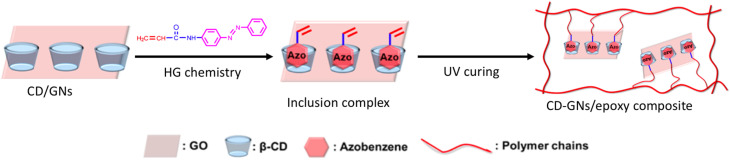
Schematic of the preparation of self-healing CD–GN/epoxy composites; reproduced from ref. 81 with permission from the American Chemical Society,^81^ copyright 2026.

In material research, the capacity of polymeric materials for self-healing is a desirable property. Multiple stimuli-responsive innate self-healing epoxy materials were constructed using HG chemistry and graphene's exceptional characteristics. β-CD was attached to graphene *via* amidation; then, acrylamidoazobenzene (AAAB) was added through HG interactions to create the CD–GNs/AAAB, which functions as a macro-cross-linker and photothermal agent. Through free radical copolymerization, the unsaturated epoxy resin was attached to the β-CD/graphene complex by ultraviolet (UV) curing, and a dynamic HG interaction between β-CD and Azo reestablished the broken bonds caused by damage. However, CD moieties in β-CD/GNs can function as noncovalent macrolinkers by incorporating Azo moieties in epoxy. By acting as a controller to unlock or lock the linking of the epoxy network and graphene, the interactions between β-CD and Azo gave the resins their inherent self-healing ability and superior mechanical properties. The effectiveness of self-healing is significantly influenced by the association constant (*K*_a_), and previous studies have shown that a higher *K*_a_ typically results in a better healing effect. Because the *K*_a_ of β-CD and Azo is approximately 10 000 M^−1^,^79^ the two compounds function well together to create a self-healing system.^80^ Out of all the documented intrinsic self-healing epoxy resins and HG self-healing systems, the epoxy composites in this work demonstrated a high healing efficiency of up to 79.2% and a tensile strength of up to 20.8 MPa under heating or near-infrared stimulation, which are comparatively excellent values. The new HG macro-cross-linking technique improves the strength and self-healing ability of plain epoxy resins. This makes it easier to use smart materials in real-life engineering. Furthermore, as graphene may be substituted with other nanomaterials, including carbon nanotubes, metallic oxides, ceramics, and quantum dots, this approach should prepare a variety of functionalities with self-healing capabilities (Fig. 6).^81^

It was reported that a photoresponsive hydrogel with strong toughness, great stretchability, and quick self-healing was cross-linked by HG interactions. The primary chain of the polymer network was functionalized with spiropyran (SP) and embellished with the Azo and β-CD pendant groups. This HG cross-linking network has excellent properties, like self-healing within 10 s, high strength capable of supporting 500 times its own weight, and 1020% elongation at break. More significantly, light irradiation at various wavelengths allowed for precise control over the creation and removal of HG interactions between Azo and β-CD, which further regulated the material's toughness, stretchability, and self-healing qualities. Irradiation of 365–440 nm light could both increase and decrease its toughness and tensile strain. However, because the hydrogel was photochromic, SP groups may have been introduced by patterning its surface with light. It was anticipated that these promising and adaptable qualities would draw attention to the materials for improved use in surface patterning, drug delivery, and tissue engineering.^82^

**Fig. 7:**
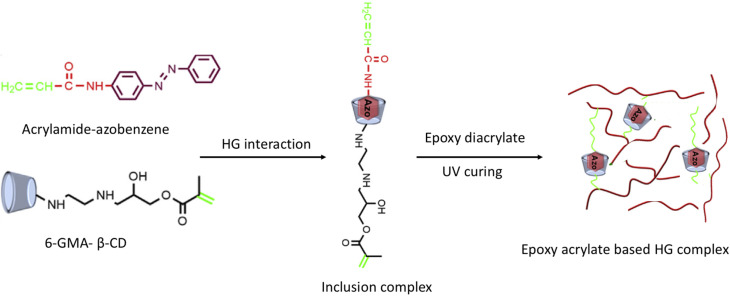
Schematic representation of the preparation of an epoxy acrylate-based HG complex; reproduced from ref. 83 with permission from Elsevier,^83^ copyright 2026.

To extend the service life, a self-healing epoxy acrylate composite with dynamic HG chemistry was devised and manufactured by Hu *et al.* (Fig. 7)*.*^83^ The self-healing epoxy acrylate film created by free radical copolymerization after the 6-glycidylmethacrylate-cyclodextrin/acrylamide Azo (6-GMA-β-CD/AAAb) was synthesized as the HG complex and interacted with epoxy diacrylate and butyl acrylate under UV curing. Because of the stronger linkages between the injured sites and improved healing, the film with the highest HG complex content had the best healing effectiveness, which was 74% after 20 min, and under moderate heating stimuli, the tensile strength of the damaged sample was recovered to 63.3% of its initial values. The produced film can be used to create smart materials, such as thermosetting composites, shape-memory materials, drug delivery systems, and smart polymers.

**Fig. 8:**
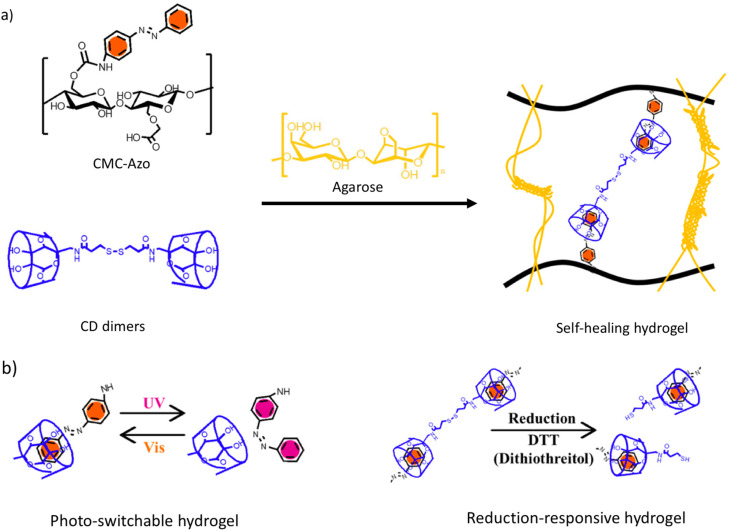
Preparation strategy of a (a) self-healing hydrogel of CMC–Azo and CD-dimers *via* HG interaction and its (b) photo-responsive and reduction-responsive characteristics; reproduced from ref. 84 with permission from Elsevier,^84^ copyright 2026.

Using Azo-grafted carboxymethyl cellulose (CMC–Azo) and β-CD dimers joined by disulfide bonds with agarose for structural support, multifunctional hydrogels were prepared in 2020 by Kim *et al.*,^84^ which includes HG complex formation (Fig. 8). In reaction to ultraviolet radiation and reducing chemicals, the resulting hydrogels demonstrated both gel–sol phase change and HG complexation-based self-healing capabilities. When exposed to external stimuli like light or a reducing agent, CMC–Azo hydrogels were able to cause a sol–gel transition. According to the *trans*–*cis* conformation shift of Azo by light and the reduction reaction of disulfide, this is caused by the HG complex formation interaction between CD-dimers and Azo in the gel. The sol–gel transition and self-healing characteristics of the hydrogels by external light were also verified by rheological research. According to the tensile and strain sweep tests, the hydrogel self-healing capacity was 79.44% and 81.59%, respectively. Moreover, a reducing agent or UV light was used to speed up the drug release from the hydrogels to 80% in 3 h. These photo-switchable, reduction-responsive, and self-healing hydrogels have the potential to be used as biomedical materials in the preparation of drug release systems based on hydrogels because of their non-cytotoxic nature.

**Fig. 9:**
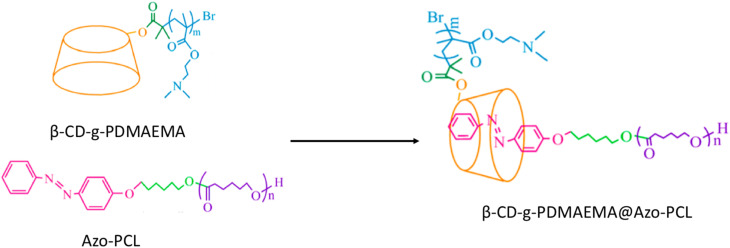
Preparation of β-CD-*g*-PDMAEMA@Azo-PCL; reproduced from ref. 85 with permission from the American Chemical Society,^85^ copyright 2026.

Supramolecular assemblies were prepared by Zhang *et al.*^85^ in 2020; these may find application as drug-controlled release vehicles. Health has consistently been a significant concern, with cancer representing one of the most substantial threats to human well-being. Although existing drug delivery systems (DDSs) have been thoroughly studied and commercialized, numerous challenges remain unresolved, including drug toxicity, side effects, and focused therapy efficiency. To overcome this challenge, a highly efficient, regulated, and targeted DDS for cancer treatment was designed and developed through HG inclusion complexation interactions. The supramolecular polymer β-CD-*g*-PDMAEMA@Azo-PCL was prepared using a host polymer, β-CD-*graft*-poly(2-(dimethylamino)ethyl methacrylate) (β-CD-*g*-PDMAEMA), and a guest polymer, Azo-modified poly(ε-caprolactone) (Azo-PCL), which has light- and pH-affected drug release properties (Fig. 9). The supramolecular assemblies ranging from spherical to irregular aggregates with varying hydrodynamic diameters formed under different pH conditions and UV-vis irradiation. The supramolecular structure effectively encapsulated doxorubicin, forming spherical core–shell drug-carrying micelles with an entrapment effectiveness of 66.1%.

**Fig. 10:**
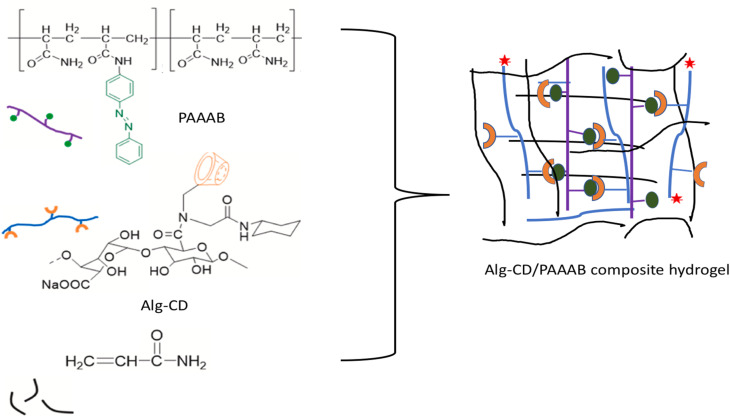
Schematic of the preparation of the Alg–CD/PAAAB composite hydrogel; reproduced from ref. 86 with permission from Elsevier,^86^ copyright 2026.

Based on the dynamic HG interaction, a highly stretchy, photocontrollable self-healing composite was prepared by utilizing alginate–CD (Alg–CD) and Azo polymer [poly(acryl-amido-azobenzene), PAABB]. The guest molecule was prepared through the copolymerization of PAAB and host Alg–CD was prepared through the Ugi reaction (Fig. 10). The mechanical strength and elasticity results showed that under dark conditions, the gels were elongated by 1330% after 48 hours, and the recovery percentage increased to approximately 90%. Even so, during exposure to UV irradiation, the separation caused between Azo and CD due to the presence of the cis structure completely stopped the self-healing capability.^86^

### 3.3 Fabrication of the self-healable supramolecular complexation of β-CD-NV

**Fig. 11:**
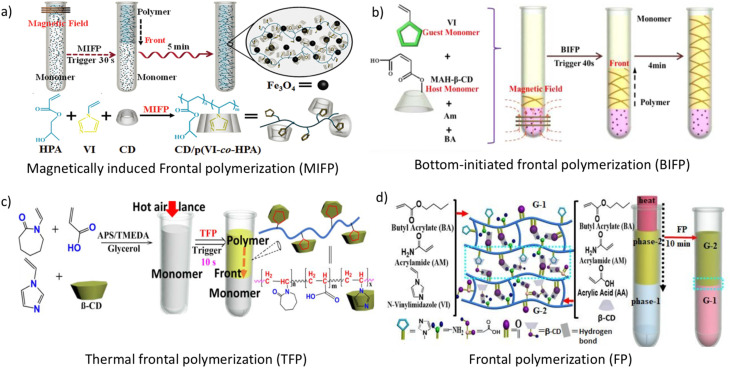
Schematic of the formation of self-healable supramolecular complexation of β-CD-NV *via* (a) MIFP method; reproduced from ref. 90 with permission from John Wiley and Sons,^90^ copyright 2026; (b) BIFP method; reproduced from ref. 91 with permission from John Wiley and Sons,^91^ copyright 2026; (c) TFP method, and (d) FP method; reproduced from ref. 92 with permission from John Wiley and Sons,^92^ copyright 2026.

In 2014, Chen's group prepared a supramolecular gel by host–guest interaction, where they used NV as a guest molecule using the magnetically induced frontal polymerization (MIFP) method, which is a coalescence of the magnetocaloric effect and frontal polymerization. Frontal polymerization (FP) is a reaction mechanism that transforms reagents into products by spreading a limited reaction zone over the entire system.^87–89^ The ensuing reaction mechanism requires no additional energy following transient stimulation with an external stimulus. By utilizing this method, they created CD/poly(vinylimidazole-*co*-hydroxypropyl acrylate) supramolecular self-healing gel by doping of Fe_3_O_4_ within 5 min (Fig. 11a).^90^ Another work was reported by the same group, where NV (VI) and β-cyclodextrin grafted carboxylic acid (MAH-β-CD) were used to prepare a self-healing hydrogel through bottom-initiated frontal polymerization (BIFP). The entire polymerization process takes place in a matter of minutes, and no additional energy is required to sustain the reaction. At room temperature, the fabricated gels showed outstanding self-healing capabilities without external stimuli, and the recovered tensile strength was 97% after 8 h due to the host–guest interaction between VI and MAH-β-CD (Fig. 11b).^91^ Using the thermal frontal polymerization process, they fabricated a copolymer utilizing β-cyclodextrin/poly(vinylimidazole-*co-N*-vinylcaprolactam-*co*-acrylic acid) (β-CD/P(VI-*co*-NVCL-*co*-AA)), which exhibited self-healing properties, high mechanical performance, and dual thermo-pH responsive activities. After 12 h, at room temperature, the hydrogel containing β-CD exhibited intrinsic self-healing without the requirement of external stimulation, which suggested that thermal frontal polymerization is more economical than photo frontal polymerization (Fig. 11c).^92^ In 2019, the frontal polymerization method was used to fabricate a self-healing gel *via* the complexation of β-CD and poly(butyl acrylate-*co*-NV-*co*-acrylamide) (BA-*co*-VI-*co*-AM) and poly(butyl acrylate-*co*-acrylic acid-*co*-acrylamide) (BA-*co*-AA-*co*-AM/β-CD), which also possesses an actuating nature. Without any external stimuli, the cut surface could mend itself at room temperature, and the repaired samples were robust enough to withstand stretching without shattering. As a reliable polymerization technique, we believe that frontal polymerization can be applied to the quick synthesis of supramolecular hydrogels.^38^

### 3.4 Fabrication of the self-healable supramolecular complexation of β-CD-N-Fc

**Fig. 12:**
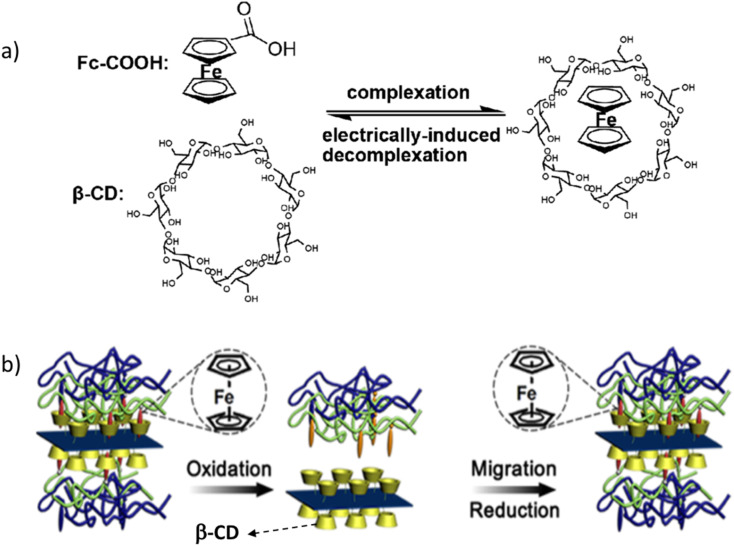
Schematic of the fabrication of (a) β-CD/Fc complexation links and a (b) (PAA/bPEI-Fc and rGO–CD) self-healable film; reproduced from ref. 95 with permission from the American Chemical Society,^95^ copyright 2026.

Electric currents can be applied quickly and easily to a variety of materials on a large scale rather than other stimuli. Many researchers are interested in electro-sensitive self-healing polymers based on different Fcs as an intriguing class of smart polymers and drug delivery devices.^38^ The first example of electrically driven self-healing polymers based on the HG interaction between β-CD and Fc has been developed by Liu *et al.* using the electrically responsive nature of the β-CD/Fc complexes, which can be used as an additive in painting products. An Fc-modified poly(glycidyl methacrylate) (PGMA-Fc) polymer chain and a difunctional β-CD molecule are used to build supramolecular networks with β-CD/Fc complexation links. Based on the decomplexation of β-CD/Fc connections, the network has demonstrated electrically driven removability and self-healing properties. The cut surface of the prepared sample was treated electrically with 1.5 V × 6 for 24 h and waited for 1 day at room temperature, followed by the thermal cure at 85 °C for 24 h to enhance the efficiency of healing.^93^ Through the HG inclusion between PEI oligomer-grafted Fc (PEI-Fc) and fatty acid (Empol-1016)-modified β-CD (Em-β-CD), a supramolecule was prepared by Zhang *et al.*,^94^ where ammonium cerium nitrate was used as an oxidant and sodium hydrogen sulfite as a reductant. The Em-β-CD/Fc complexes exhibit macromolecule behavior and have potential use as cushioning agents, which have a healing time of less than 5 min in water, and the regaining healing capacity is 73%. Sun *et al.*^95^ reported the fabrication of intrinsically healable, rGO–reinforced polymer composite films that can easily and repeatedly heal cuts within 3 h in water *via* reversible HG interaction between nanofillers and the matrix polymer films. Branched PEI-Fc (bPEI-Fc) and rGO nanosheets modified with β-CD (rGO–CD) can be assembled layer by layer to prepare the healable films (bPEI-Fc and rGO–CD) with a healing efficiency of 98% (Fig. 12).

### 3.5 Fabrication of the self-healable supramolecular complexation of β-CD-CA

**Fig. 13:**
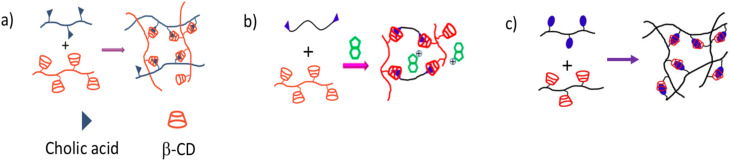
Illustration of the formation of the (a) P(DMA–CAM)/P(MDA–CDA) hydrogel; reproduced from ref. 97 with permission from the American Chemical Society,^97^ copyright 2026; (b) PDMA–CD hydrogel; reproduced from ref. 98 with permission from the American Chemical Society,^98^ copyright 2026; and (c) PVA–CA/PVA–CD hydrogel; reproduced from ref. 99 with permission from the American Chemical Society,^99^ copyright 2026.

A group of bile acids, including both primary and secondary bile acids, such as glycocholic acid, cholic acid, taurocholic acid, glycodeoxycholic acid, taurodeoxycholic acid, and deoxycholic acid, can be used as a potential guest in HG inclusion with β-CD (Fig. 13). Bile salt size and form match the β-CD cavity perfectly, as they comprise a hydrophobic tail and a steroid backbone, making them physiologically significant steroids among other guest molecules. They are essential for the digestion, absorption, and transportation of lipids, and they can make excellent building blocks for polymeric biomaterials because of their biocompatibility and functionalization potential.^96^

Thus, in the development and manufacturing of self-healing supramolecular hydrogels, there is a significant benefit of using two biological substances, β-CD and bile acids, based on G interactions.

From Table 2, we can conclude that the mechanical strength and qualities of the hydrogels depend on the cross-linking density, which can be improved by the larger mole fraction of CA as a guest molecule. The use of these low molecular weight polymers based on HG complexation provides more dominance in biodegradability.

**Table 2:** Some recent studies on self-healable materials based on the HG complexation of β-CD and CA

Host group	Guest group	Healing time	Healing efficiency	Strength of hydrogel	Ref.
β-CD-grafted poly(dimethylacrylamide) P(DMA–CDA)	CA-grafted P(DMA) P(DMA–CAM)	<1 min	*G*′ value recovered 97%, within 30 s	Depends on cross-linking density	97
PDMA–β-CD	CA–PEG–CA benzimidazole (Bzl) as a competitive guest	<1 min	97% within 30 s	CO_2_	98
Poly(vinyl alcohol) having β-CD (PVA–CD)	PVA–CA	<1 min	90%	Depends on cross-linking density	99

### 3.6 Fabrication methods of the self-healable supramolecular complexations of β-CD and miscellaneous guests

In addition to the commonly studied guest molecules, such as AD, Fc, Azo, CA, and NV derivatives, a variety of other compounds have garnered significant interest among researchers. These include benzyl, cationic alkyl chains, *N*-isopropylacrylamide, arylazopyrazole, amino acids, benzotriazole, l-methanol, phenolphthalein, and Pluronic, all of which demonstrated a notable capacity for effective interaction with the β-CD cavity, as shown in Table 3.

**Table 3:** Supramolecular complexations of β-CD and miscellaneous guest molecules

Host	Guest	Method	Healing time	Ref.
Poly(*N*-isopropylacrylamide)-*co*-6-deoxy-β-CD [P(NIPAAm-*co*-BAAm-*co*-6-AAm-β-CD)]	Poly(*N*-isopropylacrylamide) [P(NIPAAm-*co*-BAAm)]	Conventional radical polymerization	—	100
Poly-β-CD	Bromonaphthalene [BrNp]	Radical binary copolymerization	<1 min	101
Poly-CD	Poly bromonaphthalene (BrNp) and 6-thio-β-CD [6-SH-CD]	Seeding growth method	After 1 min	102
Poly(l-glutamic acid) grafted β-CD [PLGA-*g*-β-CD]	PLGA-*block*-poly(ethylene glycol)-*block*-PLGA grafted cholesterol [PLGA-*b*-PEG-*b*-PLGA-*g*-Chol]	Condensation reaction	<1 min	103
Alg-*graft*-β-CD	PEG-*block*-poly(propylene glycol)-*block*-PEG [Pluronic F108]	Covalent conjugation	Within a few seconds	104
β-CD	Pentacyclic triterpenoid glycyrrhetinic acid	Free radical polymerization	<1 min	105
β-CD	Phenolphthalein [PP]	Homogeneous radical co-polymerization	After 24 hours	106
β-CD	Cationic alkyl molecule	Radical co-polymerization	—	107
β-CD	NIPAAm	Free radical polymerization	—	108
β-CD-modified CMC	Arylazopyrazole	Photo-isomerization	—	109
β-CD	Amino acids (tyrosine, tryptophan, phenylalanine, and histidine)	Polymerization	—	110
rGO–CD	Benzotriazole [BTA]	—	—	35
β-CD-grafted chitosan [CD-*g*-CS]	l-Menthol	LbL self-assembly	<20 min	111

## 4. Comparative analysis of different β-CD host–guest systems

Although β-CD-based self-healable supramolecular systems have demonstrated remarkable versatility, their performance strongly depends on the nature of the guest molecule and the balance between binding strength and dynamic reversibility. Each guest molecule exhibits a distinct complexation constant *K*_a_ with the β-CD cavity, characterized by unique properties, like high healing rate, strong interaction, response to light and redox stimuli, in addition to mere size or steric compatibility with the β-CD cavity, as well as their hydrophobic characteristics, which have garnered significant interest from researchers. It is evident that, due to the lower binding constants of the guest molecules with the β-CD cavity, researchers have concentrated their efforts on guest molecules exhibiting higher binding constants, such as AD, Fc, and Azo (as shown in Table 4). AD exhibits the highest binding constant with β-CD (*K*_a_ ≈ 3.5 × 10^4^ M^−1^), resulting in a mechanically robust and structurally stable inclusion complex with β-CD. These systems typically demonstrate high healing efficiency and excellent load-bearing capacity. However, excessively strong inclusion may slow healing kinetics and reduce responsiveness under mild conditions. In addition, AD derivatives are synthetic and may require further biocompatibility evaluation for long-term clinical applications.^52^ Azo offers light-responsive reversibility with moderate binding affinity (*K*_a_ ≈ 10^4^ M^−1^). Its *trans*–*cis* isomerization enables spatial and temporal control of healing behavior, making it attractive for smart materials and controlled drug release. Nevertheless, photo-fatigue, limited light penetration in biological tissues, and possible photo-toxicity remain challenges for *in vivo* applications.^84,112^ Fc (17 × 10^3^ M^−1^) introduces redox responsiveness due to its reversible oxidation state. Electrically driven healing and removability are unique advantages of coatings and conductive systems. However, the oxidized Fc form loses inclusion capability, which may compromise long-term mechanical stability under oxidative physiological conditions.^113,114^ CAs provide excellent biocompatibility and biodegradability. Although their binding constant is lower (*K*_a_ ≈ 4 × 10^3^ M^−1^), they are particularly attractive for biomedical systems due to their endogenous origin. The limitation lies in their relatively lower mechanical reinforcement compared to AD systems.^115^ NV and related guests enable rapid fabrication methods, such as frontal polymerization, and provide pH-responsive behavior. However, their mechanical performance is generally moderate, and systematic comparative mechanical evaluation is still lacking.^116^ Overall, strong-binding guests enhance structural integrity, while moderate-binding guests improve dynamic adaptability and stimuli responsiveness. Therefore, the optimal host–guest pair depends on application-specific requirements, particularly in biomedical contexts where injectability, degradability, and biocompatibility must be carefully balanced.

**Table 4:** Binding constant (*K*_a_) of various inclusion complexes of β-CD with different guest molecules

Guest molecule	Binding constant (*K*_a_)	Ref.
Adamantane	3.5 × 10^4^	18
Azobenzene	10 × 10^3^	117
Ferrocene	17 × 10^3^	36
Cholic acid	4 × 10^3^	118
Bromonaphthalene	10^3^	80
Glycyrrhetinic acid	1.59 × 10^3^	105
*tert*-Butyl	1.7 × 10^2^	118
Dansyl	1.22 × 10^2^	119
Cyclohexyl	1.6 × 10^2^	118
Cyclododecyl	8.4 × 10^1^	118
Benzyl	8.4 × 10^1^	120
*N*-1-Naphthyl	1.2 × 10^2^	121
*N*-2-Naphthyl	2.7 × 10^2^	121

## 5. Biomedical applications of β-CD-based self-healable supramolecular materials

β-CD-based self-healable supramolecular materials have emerged as a versatile platform for biomedical applications, particularly in tissue engineering and regenerative medicine. Their inherent biocompatibility, enzymatic degradability into non-toxic glucose derivatives, and dynamic host–guest interactions make them highly suitable for long-term interaction with biological tissues. This self-healing capability of β-CD systems enables structural recovery under mechanical stress, which is essential for maintaining functionality in dynamic physiological environments.^122^ β-CD-based hydrogels serve as adaptable scaffolds that support cell adhesion, proliferation, and differentiation while allowing minimally invasive delivery. Their mechanical properties can be precisely tuned through HG crosslink density, enabling the fabrication of soft matrices for skin and cartilage regeneration or mechanically reinforced constructs for bone repair. Importantly, degradation rates can be engineered to match tissue regeneration timelines, eliminating the need for secondary surgical removal. A defining advantage of β-CD materials is their ability to form inclusion complexes with hydrophobic drugs, growth factors, and signaling molecules. This enables controlled and sustained release, ensuring spatially and temporally regulated delivery of therapeutic agents critical for effective tissue regeneration. Such controlled release strategies significantly enhance angiogenesis, osteogenesis, and chondrogenesis compared to conventional delivery systems.^123,124^

In wound healing, β-CD-based self-healable hydrogels provide multifunctional platforms that simultaneously address infection control, inflammation, and tissue regeneration. These systems enhance the solubility and stability of antimicrobial and anti-inflammatory agents, while their injectable and self-repairing nature allows them to conform to irregular wound geometries and withstand mechanical deformation.^125–129^ Stimuli-responsive β-CD hydrogels further enable on-demand drug release triggered by temperature, light, or natural biochemical cues.^130^ For bone regeneration, β-CD-based scaffolds and coatings improve osteoconductivity and mechanical stability while supporting sustained release of osteogenic factors. Integration with hydroxyapatite, bioactive ceramics, or conductive nanomaterials enhances load-bearing capability and promotes synergistic osteogenic and angiogenic responses. Self-healing behavior further ensures structural integrity during prolonged implantation.^131,132^ In cartilage repair, injectable β-CD hydrogels provide sustained anti-inflammatory drug delivery and mechanical reinforcement in avascular environments. Their dynamic supramolecular networks respond to mechanical loading, promoting chondrocyte activity and extracellular matrix formation. Advanced bilayer and gradient systems enable the simultaneous regeneration of cartilage and subchondral bone.^133–135^ Beyond musculoskeletal applications, β-CD-based self-healable biomaterials have shown promise in cardiac, neural, and corneal regeneration.^136,137^ Injectable hydrogels enhance cell retention and survival in myocardial tissue, while neural scaffolds support stem cell differentiation and axonal growth. Transparent β-CD hydrogels and micro-gels facilitate corneal repair by promoting epithelial cell migration without compromising optical clarity.^137,138^

Overall, β-CD-based self-healable supramolecular materials offer a powerful and adaptable toolkit for next-generation biomedical applications. Their combination of dynamic mechanical resilience, controlled bioactive delivery, and excellent biocompatibility positions them as key candidates for translational regenerative therapies (Table 5).

**Table 5:** Recent studies on β-CD-based supramolecular hydrogel materials for biomedical applications

Target	Reactants	Dopant	Materials fabrication	Importance	Ref.
Wound healing	HA, vitamin E, dopamine, and β-CD	Vitamin E	Dopamine-functionalized β-CD was chemically grafted onto hyaluronic acid, and subsequent enzymatic crosslinking-driven self-assembly yielded a β-CD-6-DA-HA film hydrogel	Accelerated wound repair in BALB/c mice by enhancing fibroblast migration, regulating cytokine levels, and improving collagen organization	139
Wound healing	β-CD, 4,7-dimethyl-2*H*-chromen-2-one (DMC) and 7-methoxy-4-methyl-2*H*-chromen-2-one (MMC)	Coumarin	Coumarin/β-CD complexes were prepared by simple mixing and subsequent lyophilization to form a scaffold	Coumarin acts as a MEK inhibitor, reducing inflammation and enhancing skin regeneration in Swiss mice	140
Wound healing	Aldehyde-modified β-CD (β-CD-CHO), CECT-AD, and CECT-ADH	Chitin	Supramolecular hydrogels are formed by preassembling β-CD-CHO with adamantan grafted carboxyethyl chitin (CECT-AD), then crosslinking with adipic dihydrazide-grafted carboxyethyl chitin (CECT-ADH)	Exhibits antimicrobial activity and accelerates skin defect healing in ICR mice	141
Wound healing	Chitosan hydrogel combined with a naringin (Nar) and β-CD inclusion nanocomplex	Naringin	Chitosan hydrogels loaded with Nar/β-CD nanocomplexes were fabricated using solvent evaporation and β-glycerophosphate crosslinking	Enhances Nar's solubility and bioavailability, promoting faster wound closure and tissue regeneration in Sprague–Dawley rats	142
Wound healing (diabetic)	Eugenol–β-CD (EG–β-CD) and CMC	Eugenol	EG is encapsulated in β-CD to form EG–β-CD complexes and is subsequently loaded into a CMC hydrogel	Controlled eugenol release regulates inflammation and angiogenesis, improving wound healing in type 1 diabetic mice	143
Wound healing (diabetic)	Thiolated c-PGA, β-CD and bilirubin	Bilirubin	Bilirubin/β-CD inclusion complexes were formed *via* solvent evaporation and integrated into a thiolated polyglutamic acid hydrogel	Enhances wound healing in both diabetic (streptozotocin-induced) and non-diabetic rat models	144
Wound healing (diabetic)	Gelatin-β-CD	Platelet-rich plasma-derived exosomes (PRP-EXOs)	Gelatin-β-CD supramolecular complexes combined with gelatin fibers and exosomes were crosslinked using genipin to improve the mechanical strength of the hydrogel	Enhances wound healing in diabetic rats by stimulating cell proliferation and migration, promoting autophagy, and reducing apoptosis under high-glucose conditions	145
Wound healing (diabetic)	Pomegranate peel extract, citric acid, β-CD, and carboxymethyl tapioca starch (CMS)	Ellagic acid derived from the pomegranate peel	Tapioca starch was carboxymethylated and combined with citric acid, β-CD, and pomegranate peel extract to produce hydrogel films	The hydrogel creates a moist, pH-responsive environment that reduces inflammation and supports cell proliferation and migration in streptozotocin-induced diabetic mouse wounds	146
Wound healing (burn)	HA, β-CD and dextran	Resveratrol, plasmid DNA encoding VEGF	UV irradiation crosslinks the modified biomaterials-HAMA, Dex-HEAA (*N*-hydroxyethyl acrylamide-modified dextran), and β-CD-PEGMA (polyethylene glycol methyl acrylate-modified β-CD) hydrogel	The resveratrol and Vascular Endothelial Growth Factor (VEGF)-loaded hydrogel promoted burn wound healing in rats by reducing inflammation and enhancing angiogenesis	147
Wound healing (bacterial infection)	β-CD-modified chitosan, AgNO_3_ and diclofenac	Diclofenac	Ag^+^ ions interact with chitosan's OH/NH_2_ groups to form an ionic hydrogel network	The hydrogel synergistically delivers Ag^+^ and diclofenac, boosting antimicrobial and anti-inflammatory effects to improve wound healing in mice	148
Wound healing (bacterial infection)	β-CD conjugated hydroxypropyl CS (HPCS-CD), poly(*N*-isopropylacrylamide) (PNIPAM), and adamantyl acrylate (ADA)	Dipotassium glycyrrhizinate (DG)	HPSC-CD and ADA form a supramolecular complex and then copolymerize with NIPAM to produce thermos-sensitive injectable hydrogels	Delivers DG, controlling infection and inflammation, thereby enhancing wound healing in Kunming mice	149
Wound healing (bacterial infection)	Gallic acid-grafted CS (CS–GA) and aldehyde-β-CD (A-β-CD)	Curcumin	CS–GA and A-β-CD crosslink to form a dynamic imine network, yielding a pH-sensitive hydrogel	Delivers curcumin, showing antimicrobial activity and enhancing wound healing in ICR mice	150
Bone	Hydroxyapatite (HAp) and β-CD-based polyurethane	HAp	β-CD, HDI, and HAp are polymerized and foamed *in situ* to form porous scaffolds	Porous structure and mechanical strength of the scaffold promote MC3T3-E1 cell adhesion and growth *in vitro*	151
Bone	Nanofibrillated cellulose (NFC) and β-CD	Raloxifene hydrochloride	Freeze-dried NFC scaffolds loaded with raloxifene–β-CD complexes were prepared to form a porous scaffold	Provided controlled raloxifene release and supported Saos-2 cell compatibility for bone regeneration	152
Guided bone regeneration	HA alkyl derivatives (HA-EDA-C_*n*_), polyvinyl alcohol (PVA) and 2-hydroxypropyl-β-CD (HP-β-CD)	Dexamethasone osteogenic	Blended materials were electrospun into nanofibers and crosslinked post-production using EDC/NHS	Modified nanofibers sustain dexamethasone release, promoting MC3T3 proliferation, differentiation, and mineralization *in vitro*	153
Vascularized bone regeneration	Methacrylated gelatin (Gel-MA) and acrylated β-CD (Ac-β-CD)	QK peptide, octacalcium phosphate (OCP)	Ac-β-CD and OCP were incorporated into gelatin and UV-photo-crosslinked to fabricate composite hydrogels	QK and OCP acted synergistically to stimulate angiogenesis and osteogenesis, significantly improving bone regeneration in rat calvarial defects	154
Bone	Gel-MA, Ac-β-CD and rGO	rGO	Gel-MA, Ac-CD, and rGO were crosslinked *via* APS/TEMED-initiated free radical polymerization to form a robust conductive hydrogel	The hydrogel enhanced bone regeneration, accelerated skull defect repair, and enabled photothermal antibacterial therapy with high biocompatibility and mechanical strength	155
Bone (osteointegration)	Pitavastatin-loaded β-CD grafted CS, gelatin and titanium substrate	Pitavastatin	A functional multilayer coating was constructed on titanium *via* LBL assembly of pitavastatin-loaded CS-β-CD and gelatin to form a hydrogel	Sustained pitavastatin release promoted osteogenic and angiogenic activities, significantly improving osseointegration in rat femoral defect models	156
Bone (femoral head necrosis)	HP-β-CD and gelatin	Bone marrow-derived mesenchymal stem cells (BMSC)	HP-β-CD and gelatin were crosslinked using 1,10-carbonyldiimidazole to form hydrogels	The HP-β-CD-gel/BMSC composite promoted osteogenesis and angiogenesis in a rat femoral head necrosis model	157
Cartilage	Gelatin, β-CD and Fe_3_O_4_ nanoparticle	BMSC	A magnetic hydrogel was prepared by GPTMS crosslinking of gelatin/β-CD with embedded Fe_3_O_4_ nanoparticles	Pulsed electromagnetic stimulation markedly enhanced MSC chondrogenesis, upregulating cartilage-specific markers and ECM formation *in vitro*	158
Cartilage (osteochondral tissue)	Methacrylated hyaluronic acid (HAMA), Gel-MA and isocyanatoethyl acrylate (AOI)-modified β-CD (β-CD-AOI2)	Kartogenin, melatonin	A biomimetic osteochondral bilayer hydrogel was fabricated *via* sequential photo-crosslinking of Gel-MA/β-CD-AOI2 and HAMA/β-CD-AOI2, encapsulating BMSCs	Site- and phase-specific release guided stem cell differentiation, enabling simultaneous regeneration of cartilage and subchondral bone in a rabbit osteochondral defect model	159
Cartilage (anti-inflammation)	Gellan gum, 6-(6-aminohexyl)amino-6-deoxy-β-CD (HCD)	Dexamethasone	HCD was grafted onto gellan gum through carbodiimide chemistry and physiologically crosslinked to form a porous injectable hydrogel	The hydrogel improved drug delivery, stimulated chondrogenesis, and reduced inflammation in rabbit cartilage defects	135
Cartilage	β-CD, CA, PLGA and chitosan	Adipose-derived stem cells (ASCs)	An injectable hydrogel was formed *via* host–guest interactions between PLGA-*co*-GM-*co*-GC and QCSG–CA, followed by *in situ* photo-crosslinking	The self-healing injectable hydrogel significantly enhanced *in vivo* cartilage regeneration, supporting minimally invasive repair strategies	134
Heart (myocardial infarction)	β-CD modified gelatin (Gel-CD) and AD modified gelatin (Gel-AD)	Induced pluripotent stem-cell-derived	Gel-CD and Gel-AD formed a self-healing injectable hydrogel *via* supramolecular host–guest crosslinking	High cell viability during injection highlights its suitability for minimally invasive cell delivery	61
Heart (ischemic myocardium)	Alg–β-CD and adamantane–GO (AD–GO)	Rat mesenchymal stem cells (MSCs)	A dual-crosslinked injectable hydrogel was prepared through Alg–CD/AD–GO host–guest assembly and calcium-mediated ionic crosslinking	The hydrogel exhibited improved mechanics, oxygen permeability, and supported MSC viability and cardiomyogenic differentiation *in vitro*	160
Heart (myocardial infarction)	Hydrazided HA (HHA), aldehyde-dextran (OD), HP-β-CD and resveratrol	Resveratrol	A lyophilized aldehyde-dextran hydrogel was crosslinked with HHA and loaded with MSCs and β-CD/resveratrol complexes	The patch enhanced MSC retention, reduced oxidative stress and fibrosis, and promoted angiogenesis in a rat myocardial infarction model	161
Nerve (spinal cord)	Gel-MA and Ac-β-CD	NSCs, OSMI-4	A UV-crosslinked Gel-MA/Ac-β-CD bio-printable hydrogel was fabricated and loaded with OSMI-4 to induce NSC neuronal differentiation	Sustained OSMI-4 release enhanced neuronal differentiation, neural regeneration, and motor recovery in a spinal cord injury model	162
Nerve (spinal cord)	Acellularized spinal cord matrix (ASCM), gelatin, Ac-β-CD, polyethylene glycol diacrylate (PEGDA) and polycaprolactone (PCL)	WAY-316606	A microfiber-embedded double-network hydrogel composite combining ASCM and G–CD–PEGDA was formed *via* thermal and UV crosslinking and reinforced with PCL microfibers	Controlled WAY-316606 release promoted neural regeneration, reduced glial scarring, and improved motor function in spinal cord injury models	163

## 6. Present and future perspectives

β-CD-based self-healable supramolecular materials are assured to play a pivotal role in the development of next-generation smart and sustainable material systems. Despite significant progress in β-CD-based supramolecular self-healing systems, several challenges remain. A key limitation lies in balancing binding strength and dynamic reversibility; systems with high *K*_a_ values offer superior mechanical strength but may exhibit slower healing kinetics, while weaker complexes compromise structural integrity. Furthermore, most reported systems lack standardized mechanical evaluations, making cross-study comparisons difficult. Long-term *in vivo* stability, fatigue resistance, and translational scalability also remain underexplored. Future research should prioritize the precise molecular engineering of HG interactions to achieve tunable healing kinetics, improved mechanical strength, and long-term fatigue resistance under repeated mechanical stress. The incorporation of multi-stimuli responsiveness such as pH, temperature, light, and redox triggers into β-CD HG architectures further enables the design of adaptive materials for advanced biomedical applications.

Future investigations should focus on the behavior of β-CD-based systems within complex biological environments, including their long-term stability, degradation pathways, and the impact of these processes on therapeutic performance. This functional efficiency of β-CD materials can be further enhanced through the formation of composites with biopolymers such as alginate or chitosan, as well as the incorporation of metal ions (Ca^2+^ and Ag^+^) to improve mechanical resilience under physiological conditions. Host–guest interactions involving specific guest molecules, such as adamantane for structural stability or azobenzene for light-responsive healing, provide additional opportunities for dynamic functionality. Moreover, the integration of antimicrobial agents, including silver nanoparticles or essential oils, offers protection against biofilm formation. The development of composite architectures and stimuli-responsive linkages, such as pH-sensitive acylhydrazone bonds, enables self-repair and site-specific drug release, particularly in inflamed tissues or bone defects. Collectively, these advances support the creation of biomimetic materials that resemble the extracellular matrix, promoting cell growth while maintaining high self-healing efficiency and durability for long-term clinical use. For biomedical coating applications, β-CD-based supramolecular systems are increasingly optimized through dual-crosslinking strategies that combine reversible host–guest interactions with permanent or stimuli-responsive covalent bonds. This approach allows for a balance between mechanical robustness and rapid healing capability. The use of poly-β-CD architectures and nano-reinforcements, such as functionalized nanosheets, can further enhance crosslinking density, mechanical integrity, and environmental resistance.

From a translational perspective, improving scalability, processing compatibility, and long-term stability remains critical for real-world deployment. The combination of β-CD-mediated supramolecular interactions with covalent or hybrid crosslinking strategies may overcome current limitations related to mechanical strength and environmental sensitivity. Furthermore, expanding the application scope toward multifunctional systems such as self-healing materials with antimicrobial, antioxidant, or controlled-release capabilities will significantly enhance their practical value. Overall, continued interdisciplinary efforts in molecular design, materials engineering, and application-driven testing will accelerate the transition of β-CD-based self-healable supramolecular systems from laboratory research to industrial and biomedical implementation.

## 7. Conclusion

β-CD-based supramolecular materials fabricated through host–guest interaction possess significant qualities, including shape memory, self-degradability, and autonomous self-healing. The superior structure of CD, with its hydrophobic interior and hydrophilic exterior, enables it to easily form complexes through incorporation with a variety of impartial guest molecules. AD, when acting as a guest molecule, can form a stable inclusion complex with the β-CD cavity and exhibit a significantly higher association constant with the β-CD cavity in comparison to other guest molecules. The reduced form of Fc can be accommodated within the β-CD cavity; however, its oxidized form is excluded due to its hydrophilic characteristics. The Fc and β-CD complexes, which respond effectively to straightforward and beneficial electrochemical stimuli, have garnered considerable interest among researchers. Additionally, the Azo and β-CD are well suited for light-responsive applications, as they allow for the reversible inclusion and exclusion of the Azo moiety from the β-CD cavity under UV-vis stimuli. Furthermore, hydrogels created through the inclusion of the complexation of biocompatible guests, such as CA and glycyrrhetinic acid, with β-CD present promising opportunities for biomedical applications like bio-inspired materials and drug delivery, as well as 3D printing, coating, and other stimuli-responsive systems.

## Conflicts of interest

There are no conflicts to declare.

## Data Availability

No primary research results, software or code have been included and no new data were generated or analysed as part of this review.
